# Synthesis, Characterization, and Photocatalytic Activity of Magnetically Separable Fe_3_O_4_@SiO_2_@ZnO–Ag Composite Photocatalyst

**DOI:** 10.1002/gch2.202400093

**Published:** 2024-07-17

**Authors:** Hakan Kiziltas, Taner Tekin, Derya Birhan, Derya Tekin

**Affiliations:** ^1^ Department of Chemical Engineering Ataturk University Erzurum 25240 Turkey; ^2^ Department of Metallurgy and Materials Engineering Ataturk University Erzurum 25240 Turkey

**Keywords:** acid blue 161, core–shell structure, Fe_3_O_4_@SiO_2_@ZnO–Ag, magnetic separation, photocatalytic degradation, ZnO nanoparticles

## Abstract

Magnetically separable Fe_3_O_4_, Fe_3_O_4_@SiO_2_, Fe_3_O_4_@SiO_2_@ZnO, and Fe_3_O_4_@SiO_2_@ZnO–Ag composites are synthesized using hydrothermal and wet chemistry methods. The samples obtained are characterized in terms of morphology, composition, optical, and magnetic properties using TEM, SEM‐EDS, XRD, FTIR, VSM, XPS, and UV–vis, and the photodegradation of Acid Blue 161 dye under UV irradiation is investigated. As a result of SEM and TEM analyses, the diameters of Fe_3_O_4_, Fe_3_O_4_@SiO_2_, Fe_3_O_4_@SiO_2_@ZnO, and Fe_3_O_4_@SiO_2_@ZnO–Ag composites are determined as 210, 220, 230 and 235 nm, respectively. The magnetic properties of the samples are determined by VSM analysis. In VSM analyses, magnetization saturation values of Fe_3_O_4_, Fe_3_O_4_@SiO_2_, Fe_3_O_4_@SiO_2_@ZnO, and Fe_3_O_4_@SiO_2_@ZnO–Ag composites are determined as 81, 64, 41 and 20 emus × ^g−1^, respectively. In XRD analysis, the face‐centered cubic structure of Fe_3_O_4_ particles and the hexagonal wurtzite structure of ZnO are determined and it is determined that they are compatible with standard diffraction cards. According to UV–Vis analysis, *E*_g_ values for Fe_3_O_4_, Fe_3_O_4_@SiO_2_, Fe_3_O_4_@SiO_2_@ZnO, and Fe_3_O_4_@SiO_2_@ZnO–Ag composites are found as 1.3, 1.68, 2.21, and 2.15 eV, respectively. Among the photocatalysts prepared, Fe_3_O_4_@SiO_2_@ZnO–Ag composite Acid Blue 161 shows superior removal and recyclability against photodegradation of the dyestuff.

## Graphical abstract

Silver‐decorated Fe_3_O_4_@SiO_2_@ZnO composite, which can be recycled and used in wastewater treatment, is synthesized using multi‐step chemical methods. The basis of the study is reusable and environmentally friendly core–shell structured photocatalysts. TEM, SEM‐EDS, XRD, FTIR, XPS, VSM, and UV–vis analyses are performed to characterize the photocatalyst. Photocatalytic activity studies are carried out with Acid Blue 161 dyestuff.

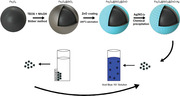

## 1 Introduction

With the rapid development of modern science and technology, there are developments in various industrial fields. As a result of these developments, water pollution is becoming an increasingly serious problem.^[^ ^1^ ^]^ In recent years, good design, morphology and synthesis methods of high‐quality composite materials have attracted great attention. In particular, in semiconductor photocatalysts used for wastewater treatment, photocatalytic activity is significantly affected by combining different components in the photocatalysis field.^[^ ^2^ ^]^ Polluted water released into the environment causes serious damage to both human health and the biological environment. Synthesized dyes are widely used in the cosmetic, plastic, and textile industries.^[^ ^3^ ^]^ The wastewater, which contains dye causes serious pollution in the environment due to its high toxicity and extreme chemical oxygen requirement.^[^ ^4^ ^]^ As an organic dye, Rhodamine B, Congo Red, Orange G, Acid Blue 161, Methyl Orange, and Methylene Blue cause serious pollution in industrial wastewater due to their widespread applications.^[^ ^5^, ^6^ ^]^ In recent years, methods such as adsorption, membrane separation, sedimentation, filtration, and biodegradation have been explored to clean wastewater. Photocatalytic purification is an important method for removing organic pollutants in wastewater because of its advantages such as being environmentally friendly, cost‐effective, and high efficiency. Since 1972, photocatalysis has been widely studied to break down organic pollutants and convert them into more stable products. Among many semiconductor metal oxides such as TiO_2_,^[^ ^7^ ^]^ CuO,^[^ ^8^ ^]^ MoS_2_,^[^ ^9^ ^]^ ZnO,^[^ ^10^ ^]^ CdS,^[^ ^11^ ^]^ MnO_2_,^[^ ^12^ ^]^ and ZnFe_2_O_4_,^[^ ^13^ ^]^ ZnO is the most widely used photocatalyst due to its special electronic band structure. ZnO, a semiconductor photocatalyst, is preferred over TiO_2_ photocatalyst due to its ease of access, high biocompatibility, thermal stability, piezoelectric constant, and superior electron mobility. In addition to these areas, it is used as a catalyst in photocatalytic decomposition.^[^ ^14^ ^]^ Zinc oxide(ZnO) has good photocatalytic properties, such as good chemical and mechanical stability, non‐toxic, low cost due to its too much in nature, and highlight sensitivity.^[^ ^2^ ^]^ However, ZnO has a wide bandgap of (3.37 eV at room temperature) and is not very effective in terms of photocatalytic activity due to its high recombination rate of photon generate carriers.^[^ ^15^ ^]^ Among all nano‐structured materials, magnetite Fe_3_O_4_ nanoparticles are widely used as magnetic adsorbents due to their strong magnetic property, low cost, high absorption ability, and unique properties.^[^ ^16^ ^]^ However, pure Fe_3_O_4_ tends to oxidize, leading to the formation of magnetite (Fe_2_O_3_) with different magnetic and chemical properties. Since the separation and adsorption of heavy metals from the solution is difficult, coating with stable materials such as silica and nanometal oxides, which act as sorbent, is performed to solve this problem.^[^ ^17^ ^]^ There are many studies available to increase the photocatalytic activity of the semiconductor photocatalyst. Among the substances mentioned above, SiO_2_ is preferred as the functional shell for the Fe_3_O_4_ core due to its high dispersion in water, many hydroxyl groups formed on its surface, and stable chemical properties. Doping metal ions into the Fe_3_O_4_@SiO_2_ core‐shell structure covered with ZnO semiconductor causes the optical band gap to shift to the visible region, controlling surface defects and increasing fluorescence. Silver (Ag) doping to ZnO contributes to the change of chemical or physical properties and optical absorption of ZnO. Ag used due to its superior electrical conductivity and affordability, can increase the performance of the photocatalytic activity by preventing the recombination of electron‐hole pairs of semiconductor photocatalysts.Wang et al. synthesized Fe_3_O_4_@SiO_2_@ZnO–Ag composite and they examined the removal of RhB dye under UV light. As a result of the experiments, it was determined that the composite removed 98.1% of RhB within 100 min.Bavarsiha et al. synthesized Fe_3_O_4_@SiO_2_@ZnO composite and studied the removal of methylene blue. Within 180 min, the composite removed 62% of the dye.Xue et al. synthesized iron oxide@HTCC using the co‐hydrothermal treatment method. In the 140 min photocatalytic experiment results, the sample removed over 95% of the MB dyestuff. In a study by Esfandiari et al., they synthesized Fe_3_O_4_@SiO_2_@TiO_2_ composite and used it in the removal of E.coli bacteria. As a result of the experiments, it was determined that the composite showed high bacterial adsorption and damaged the cell membrane of bacteria. In addition, it was revealed that the Fe_3_O_4_@TiO_2_ composite did not harm bacterial cells, since the SiO_2_ shell prevented the growth of the TiO_2_ crystal.

Silver‐decorated Fe_3_O_4_@SiO2@ZnO composite, which can be recycled and used in wastewater treatment, was synthesized using multi‐step chemical methods. The study is based on the production of environmentally friendly, easily recyclable, magnetic, and reusable photocatalysts. ZnO and Ag nanoparticles used to design core‐shell structures are attached to the surface of the Fe_3_O_4_@SiO_2_ shell structure. The amorphous SiO2 shell was used to bond functional materials, prevent the migration of photoconductor carriers from the semiconductor photocatalyst to the magnetic core, prevent the contact of Fe_3_O_4_ with the solution, and increase the surface area of the photocatalyst. Fe_3_O_4_@SiO_2_@ZnO–Ag composite was used to remove Acid Blue 161 (AB161) organic dye in wastewater. Additionally, the effect of scavengers such as KI, BQ, IPA, and KBrO3 on the photocatalytic degradation process was examined. Since the reaction takes place independent of temperature and in the presence of a catalyst, the conversion efficiency is quite high. To examine the structural properties of synthesized composites TEM, SEM‐EDS, XRD, FTIR, VSM, and XPS analyses were performed, and UV‐Vis analysis was used to examine the optical properties of synthesized composites.

## 2 Results and Discussion

**Figure 1:**
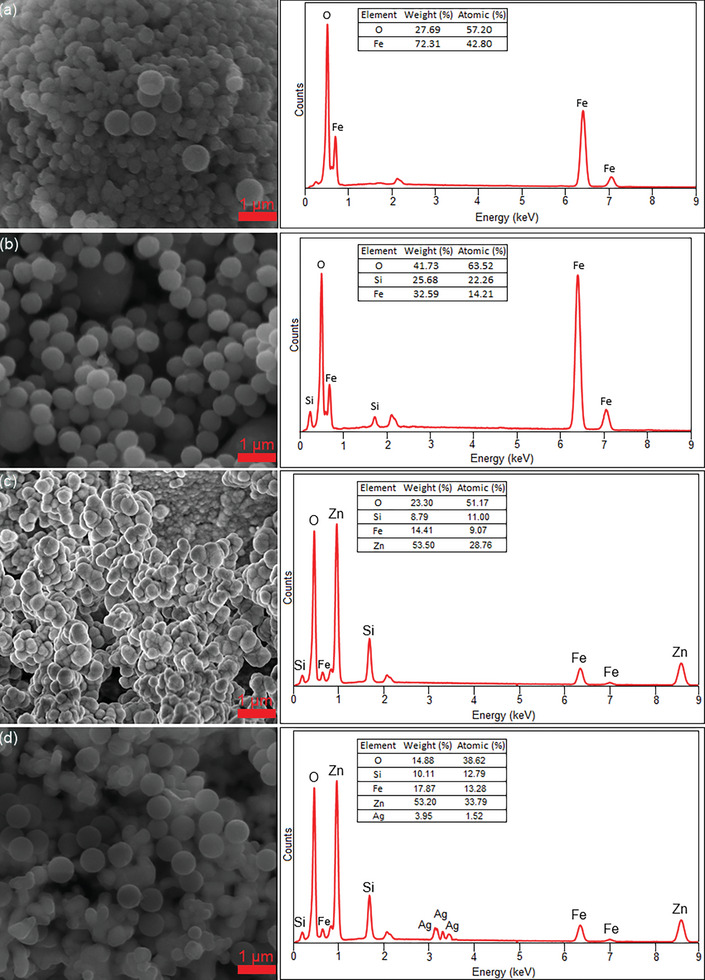
SEM‐EDS images of Fe_3_O_4_, Fe_3_O_4_@SiO_2_, Fe_3_O_4_@SiO_2_@ZnO, and Fe_3_O_4_@SiO_2_@ZnO–Agcomposites.

The SEM and EDS analysis results of the Fe_3_O_4_, Fe_3_O_4_@SiO_2_, Fe_3_O_4_@SiO_2_@ZnO, and Fe_3_O_4_@SiO_2_@ZnO–Ag composites were given in **Figure** **1**. In the first step, the solvothermal method was used to synthesize Fe_3_O_4_ particles. The Fe_3_O_4_ particles shown in Figure 1a have a diameter of ≈210 nm. Fe_3_O_4_ particles have a spherical, rough surface, and opaque structure. The Fe_3_O_4_@SiO_2_ composite shown in Figure 1b was synthesized using the Stöber method, and the diameter of the Fe_3_O_4_@SiO_2_ composite is ≈220 nm. Fe_3_O_4_ particles coated with SiO_2_ became a little more transparent. Since the SiO_2_ shell is inhibiting charge transfer between the Fe_3_O_4_ core and the catalyst shell, it may a negative effect on photocatalytic activity. As seen in Figure 1c, the Fe_3_O_4_@SiO_2_ composite was coated with ZnO using the sol–gel method. Fe_3_O_4_@SiO_2_@ZnO composite is spherical and semi‐rough. As a result of the coating of Fe_3_O_4_@SiO_2_ composite with ZnO, agglomeration occurred and caused deformations in the particles. It was calcined at 200 °C for 2 h to obtain crystalline ZnO nanoparticles. The Fe_3_O_4_@SiO_2_@ZnO composite has a diameter of about 230 nm and is larger than the Fe_3_O_4_@SiO_2_ composite. As shown in Figure 1d, the Fe_3_O_4_@SiO_2_@ZnO–Agcomposite does not show a homogeneous distribution similar to Fe_3_O_4_@SiO_2_@ZnO. Ag coating increased agglomeration and caused an increase in deformations. The Fe_3_O_4_@SiO_2_@ZnO–Ag composite has a diameter of ≈235 nm and is larger than the Fe_3_O_4_@SiO_2_@ZnO composite. EDS analysis proved the presence of Fe, O, Si, Zn, and Ag.

**Figure 2:**
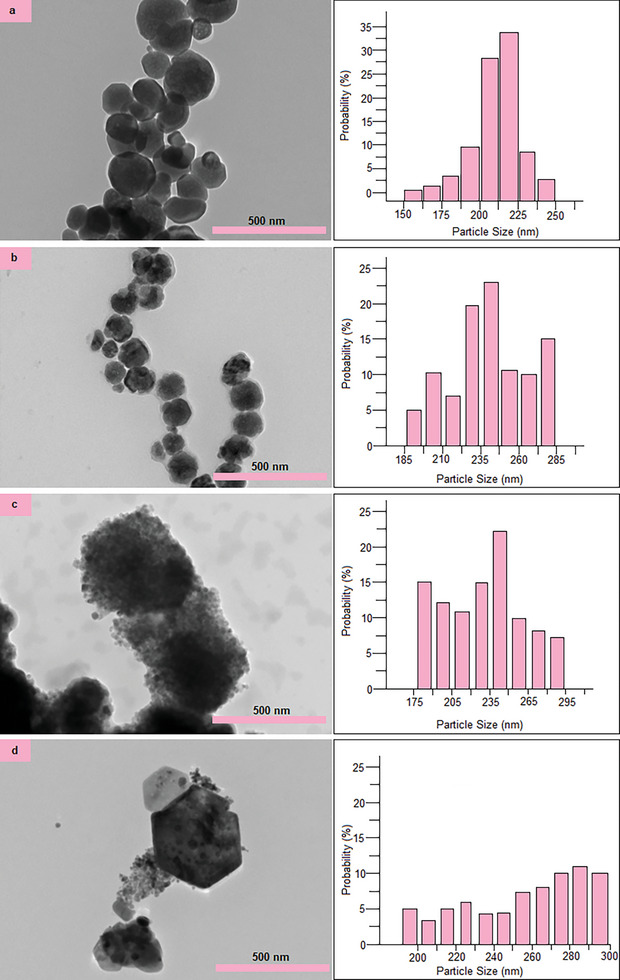
TEM images of a) Fe_3_O_4_, b) Fe_3_O_4_@SiO_2_, c) Fe_3_O_4_@SiO_2_@ZnO, and d) Fe_3_O_4_@SiO_2_@ZnO–Ag in500 nm scales and size distribution histograms of thecomposites.

The TEM analysis results of the Fe_3_O_4_, Fe_3_O_4_@SiO_2_, Fe_3_O_4_@SiO_2_@ZnO, and Fe_3_O_4_@SiO_2_@ZnO–Ag composites are given in **Figure** **2**. The synthesized Fe_3_O_4_ magnetic core has a regular spherical shape and a rough surface with an approximate diameter of 210 nm. In a study conducted in the literature, few of the small‐sized nanocrystals have a spherical shape, while most of the large‐sized nanocrystals have a cubic shape.^[^ ^18^ ^]^ As shown in Figure 2a, Fe_3_O_4_ particles have a granular structure and show that the Fe_3_O_4_ particles are formed by the combination of small diameter Fe_3_O_4_ nanoparticles.

As shown in Figure 2b, the Fe_3_O_4_@SiO_2_ composite has a smooth surface and a diameter of ≈220 nm, and similar morphological properties as pure Fe_3_O_4_ particles. In the Fe_3_O_4_@SiO_2_ composite, the black color represents the Fe_3_O_4_ core and the gray color represents a core–shell structure consisting of a SiO_2_ shell. In the Fe_3_O_4_ magnetic core covered with an amorphous SiO_2_ shell, oxidation of nanoparticles and their bonds with other functionalized materials are prevented due to SiO_2_. The SiO_2_ layer coated on Fe_3_O_4_ particles protects the nanoparticles from chemical corrosion and photo corrosion. In addition, the SiO_2_ shell is very important in preventing photodissolution. However, the thickness of the SiO_2_ shell must be adjusted well. If the shell is too thick or thin, the magnetization phenomenon decreases and the recovery of magnetic particles becomes difficult. And all of these reduce photocatalytic activity. To control the thickness of the SiO_2_ shell, TEOS concentration, reaction time, alcohol, temperature, and water are important factors. The Stöber method was used to obtain the SiO_2_ shell with ideal thickness. The shell formed around Fe_3_O_4_ particles is approximately 5 nm. The thin SiO_2_ shell is visible in the TEM image in Figure 2b.

As shown in Figure 2c, the rough surface of the Fe_3_O_4_@SiO_2_ composite is coated with a homogeneous ZnO layer and there is no obvious agglomeration. The SiO_2_ layer cannot be observed clearly due to ZnO particles. The TEM image showed that ZnO accumulated around the magnetic core instead of homogeneous nucleation. The average diameter of Fe_3_O_4_@SiO_2_@ZnO composite is ≈230 nm. As shown in Figure 2d, synthesized Fe_3_O_4_@SiO_2_@ZnO–Ag composite has a homogeneous distribution like the Fe_3_O_4_@SiO_2_@ZnO composite, and Ag nanoparticles are homogeneously dispersed on the surface of Fe_3_O_4_@SiO_2_@ZnO. Ag nanoparticles appear as granular structures on the Fe_3_O_4_@SiO_2_@ZnO–Ag surface. Particle diameter distribution analysis revealed the average diameter of the Fe_3_O_4_@SiO_2_@ZnO–Ag composite to be ≈235 nm.

**Figure 3:**
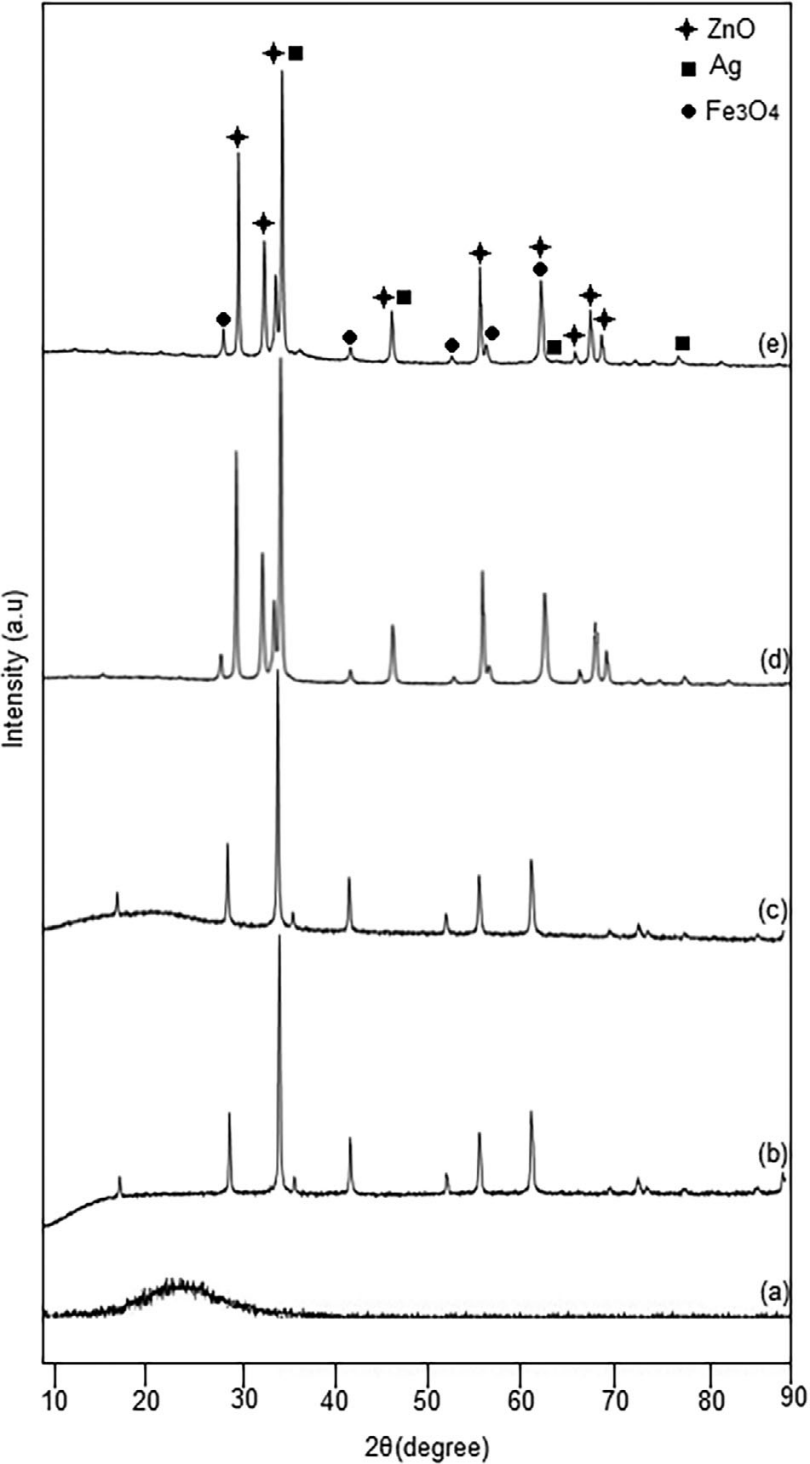
XRD diagram of a)SiO_2_, b)Fe_3_O_4_, c)Fe_3_O_4_@SiO_2_, d)Fe_3_O_4_@SiO_2_@ZnO, and e) Fe_3_O_4_@SiO_2_@ZnO–Ag composites.

**Figure** **3** shows the XRD diagram showing the prepared samples, purity, composition, and crystallinity. The Debye‐Scherrer formula was used to calculate the average diameter of the synthesized Fe_3_O_4_, Fe_3_O_4_@SiO_2_, Fe_3_O_4_@SiO_2_@ZnO, and Fe_3_O_4_@SiO_2_@ZnO–Ag composites.

(1) $$D_{\mathrm{XRD}}=\frac{k.\lambda }{\beta .\cos\theta }$$

D_XRD_ = average size of particles; θ = diffraction angle; *k* = Scherrer constant; λ = X‐ray wavelength (Cu‐Kα = 0.154 nm) and β = corresponds to the diffraction peak observed at half maximum of a diffraction peak. According to the Debye‐Scherrer formula, the average diameters of Fe_3_O_4_, Fe_3_O_4_@SiO_2_, Fe_3_O_4_@SiO_2_@ZnO, and Fe_3_O_4_@SiO_2_@ZnO–Ag composites were calculated as 207, 212, 238 and 238.8, respectively. Figure 3a belongs to amorphous SiO_2_. It is understood that the synthesized SiO_2_ nanoparticles do not have a crystal structure and therefore do not show a sharp peak. As shown in Figure 3b, Fe_3_O_4_ particles give diffraction peaks at 30.08°, 35.40°, 43.02°, 53.38°, 56.86°, and 62.46°. These diffraction peaks represent the face‐centered cubic structure of magnetite in the planes (220), (311), (400), (422), (511), and (440) (JCPDS No: 19–0629) respectively.^[^ ^19^ ^]^ Also, no impurity was observed on Fe_3_O_4_ particles. As shown in Figure 3c, Fe_3_O_4_ particles coated with an amorphous SiO_2_ layer have the same structure as pure Fe_3_O_4_. As shown in Figure 3d, surface‐centered cubic magnetite peaks were observed in the Fe_3_O_4_@ SiO_2_@ZnO composite, so we can say that Fe_3_O_4_ nanocrystals do not change their phase. ZnO phase with hexagonal wurtzite structure, corresponding to angles of 31.97°, 36.45°, 47.79°, 57.28°, 62.94°, and 68.37°, (100), (101), (102), (110), (103) and (112) shows the diffraction peaks in the planes (JCPDS No:36−1451).^[^ ^20^ ^]^ As shown in Figure 3e, the synthesized Fe_3_O_4_@SiO_2_@ZnO–Ag composite overlaps with the cards of Fe_3_O_4_ (JCPDS No: 19–0629) and ZnO (JCPDS No: 36–1451). Apart from these peaks, the diffraction peaks at 37.90⁰, 44.10⁰, 64.30⁰, and 77.20⁰ can be easily indexed to the surface‐centered‐cubic structure of Ag (JCPDS No. 04–0783).^[^ ^1^ ^]^ This shows that crystallized Ag nanoparticles are deposited in the outer shell.

**Figure 4:**
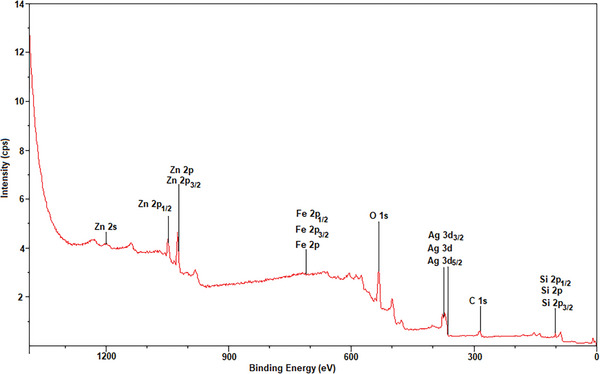
XPS spectrum of Fe_3_O_4_@SiO_2_@ZnO–Ag composite.

XPS analysis was performed to examine the elemental and chemical structures of the prepared core–shell composites and the results of Fe_3_O_4_@SiO_2_@ZnO–Ag were compared with that of Fe_3_O_4_@SiO_2_@ZnO. **Figure** **4** shows the XPS analysis of composites from 0 to 1200 eV. The peaks of the composites shown in Figure 4 are assigned to Si, Zn, O, Ag, Fe, and C elements. The C 1s peak is attributed to the pollution present in the structure. Since the Fe_3_O_4_ magnetic core is far away from the surface, the Fe 2p peak has weak binding energy. The high‐resolution XPS spectrum of Zn 2p is shown in Figure 4. Accordingly, the two peaks seen at binding energies at 1021.0 and 1044.1 eV can be indexed to Zn 2p_3/2_ and Zn 2p_1/2_, respectively. XPS results confirm that the element Zn accumulates as Zn^+2^ on the surface.^[^ ^21^ ^]^ The density of the peak of Zn 2p decreases by the deposition of Ag nanoparticles on the surface of ZnO. The XPS spectrum of the Ag 3d peak is shown in Figure 4. The two peaks at 366.7 and 372.7 eV can be indexed to AgO to Ag 3d_5/2_ and Ag 3d_3/2_, respectively. Since the binding energy of Ag 3d_5/2_ is lower than that of zero valence Ag (368.2 eV), we can see that Ag 3d_5/2_ transitions to lower binding energies at XPS peaks.^[^ ^22^ ^]^

**Figure 5:**
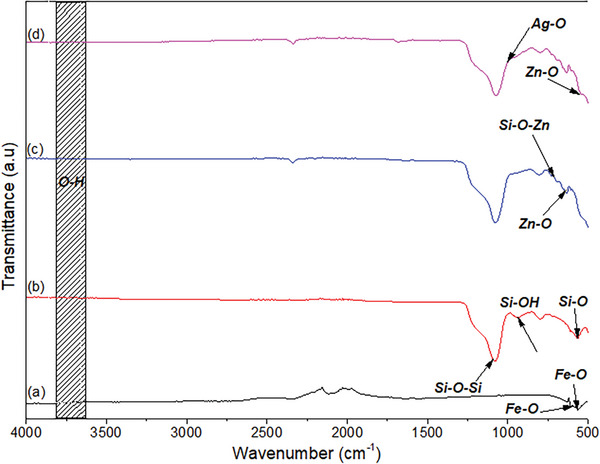
FTIR spectrum of a) Fe_3_O_4_, b) Fe_3_O_4_@SiO_2_, c) Fe_3_O_4_@SiO_2_@ZnO, and d) Fe_3_O_4_@SiO_2_@ZnO–Ag nanocomposites.

**Figure** **5**, shows the FTIR spectrum of Fe_3_O_4_, Fe_3_O_4_@SiO_2_, Fe_3_O_4_@SiO_2_@ZnO, and Fe_3_O_4_ @SiO_2_@ZnO–Ag composites in the between 4000 and 500 cm^−1^ range. As shown in Figure 5a, the Fe–O absorption band gave a dense peak at 568 cm^−1^ due to Fe‒O bonds in the tetrahedral lattice structure of Fe_3_O_4._^[^ ^23^ ^]^ Most of the time, the Fe─O bond vibrates at a wavelength of 609 cm^−1^, but due to the crystal structure and smaller particles, the vibratory band may shift to higher regions.^[^ ^24^ ^]^ As shown in Figure 5b, the peaks seen at 510 and 940.3 cm^−1^ are due to the bending vibration of the Si─O bond and the stress vibration of the Si─OH bond, respectively. Asymmetric tensile vibration of the Si─O─Si bond was observed at the peak at 1088.8 cm^−1^, so we can say that SiO_2_ was successfully coated on the surface of Fe_3_O_4_ particles.^[^ ^25^ ^]^ As shown in Figure 5c, the peak at 633 cm^−1^ corresponds to the Zn–O stretching vibration in the ZnO lattice structure, and the peak in the range 710–712 cm^−1^ corresponds to the Si–O–Zn vibration.^[^ ^26^ ^]^ The synthesized Fe_3_O_4_@SiO_2_@ZnO–Ag composites show the same band values as the Fe_3_O_4_@SiO_2_@ZnO composite. For Ag nanoparticles, the peak at 3730 cm^−1^ is because of the O–H stretching vibration of the adsorbed water. Peaks at 550–1000 cm^−1^ are attributed to Zn─O, and Ag─O bonds. Figure 5d shows that there is no clear absorption peak for AgO, but affects the shape and density of the Fe_3_O_4_@SiO_2_@ZnO composite.^[^ ^27^ ^]^

**Figure 6:**
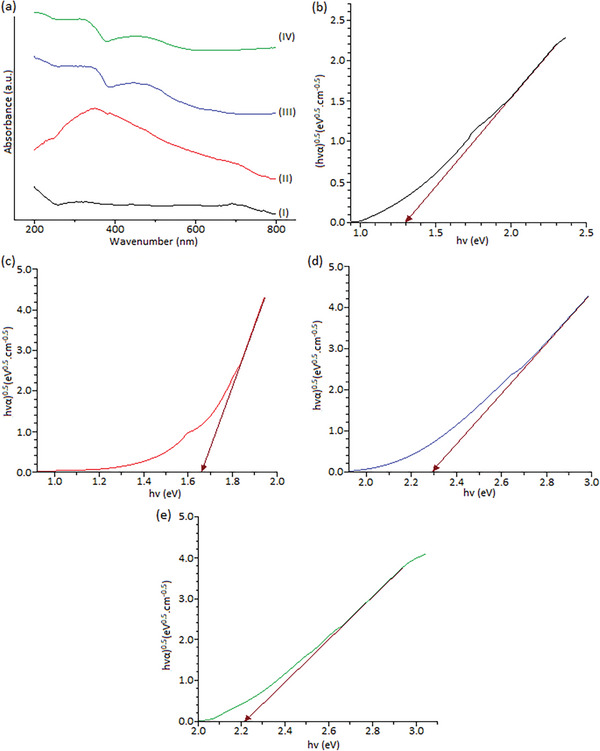
(a) UV–vis absorption spectra of Fe_3_O_4_, Fe_3_O_4_@SiO_2_, Fe_3_O_4_@SiO_2_@ZnO and Fe_3_O_4_@SiO_2_@ZnO–Ag nanocomposites, (b‐I) Fe_3_O_4_, c‐II) Fe_3_O_4_@SiO_2_, (d‐III) Fe_3_O_4_@SiO_2_@ZnO and (e‐IV) Tauc plot of Fe_3_O_4_@SiO_2_@ZnO–Ag composites.

**Figure** **6**a shows the UV–vis absorption spectra and the Tauc plot of Fe_3_O_4_, Fe_3_O_4_@SiO_2_, Fe_3_O_4_@SiO_2_@ZnO, and Fe_3_O_4_@SiO_2_@ZnO–Ag composites. The Tauc Equation is used to determine the optical band gap energies of composites and is defined as;

(2) $$\alpha h\upsilon =A{\left(h\upsilon -E_{g}\right)}^{n}$$

where A is the proportionality constant, *α* is the absorption coefficient, *hʋ* is the photon energy, *E*_g_ is the bandgap energy, and *n* is the frequency of the photon (The value *n* = 2 is for indirect permitted transitions).^[^ ^28^ ^]^ Figure 6b–e shows the plot of (*αhυ*)^2^ versus photon energy (*hυ*) for composites. The value of the energy gap is determined by extrapolating the linear region plotted against (*αhυ*)^2^ on the *y*‐axis of (*hυ*) on the x‐axis. The found *E*_g_ values for the Fe_3_O_4_, Fe_3_O_4_@SiO_2_, Fe_3_O_4_@SiO_2_@ZnO, and Fe_3_O_4_@SiO_2_@ZnO–Ag composites are 1.3, 1.68, 2.21, and 2.15 eV respectively. The bandgap values obtained for Fe_3_O_4_ nanoparticles vary between 1.16 and 2.2 eV. The main reason why the values change in this range is due to the thickness of the thin Fe_2_O_3_ layer formed on the surface of the nanospheres during synthesis. The band gap of Fe_3_O_4_ nanoparticles was determined as 1.3 eV. The forbidden energy band gap found for Fe_3_O_4_@SiO_2_ has a value of 1.68 eV. The band gap energy is expected to increase compared to Fe_3_O_4_, but the increase is minimal. The main reason for this is that the increase in particle size causes the band gap of the material to decrease and the absorption peak to shift toward the higher wavelength side. Coating the ZnO semiconductor on the surface of the Fe_3_O_4_@SiO_2_ composite, which has a bandgap of 1.68 eV, proves the accuracy of the value found for the Fe_3_O_4_@SiO_2_@ZnO composite, as it increases the bandgap. The bandgap of the Fe_3_O_4_@SiO_2_@ZnO composite was calculated to be 2.2 eV. Since Ag nanoparticles are good conductors, they are frequently used in optical studies. The band gap is predicted to decrease by coating Ag nanoparticles on the surface of the Fe_3_O_4_@SiO_2_@ZnO composite, which is found to be 2.2 eV. The bandgap of the Fe_3_O_4_@SiO_2_@ZnO–Ag composite was determined as 2.15 eV. The absorption intensity of Fe_3_O_4_ particles in the range of 200–800 nm gradually decreases and no obvious absorption peak is observed. After the surface of Fe_3_O_4_ particles was coated with SiO_2_, the absorption peak was observed at 348 nm. Confinement of small particle‐free electrons to the metal core affects the peak width of Fe_3_O_4_ covered with a SiO_2_ shell. This shows that Fe_3_O_4_@SiO_2_ composite was formed. With the precipitation of Ag nanoparticles on the surface, the maximum absorption peak, which is the characteristic feature of Ag nanoparticles, appears at approximately 454 nm. As a result, it is understood that since Ag nanoparticles prevent the recombination of electron‐hole pairs and provide effective charge separation, they will increase the photocatalytic activity by precipitating on the surface of the Fe_3_O_4_@SiO_2_@ZnO composite.

**Figure 7:**
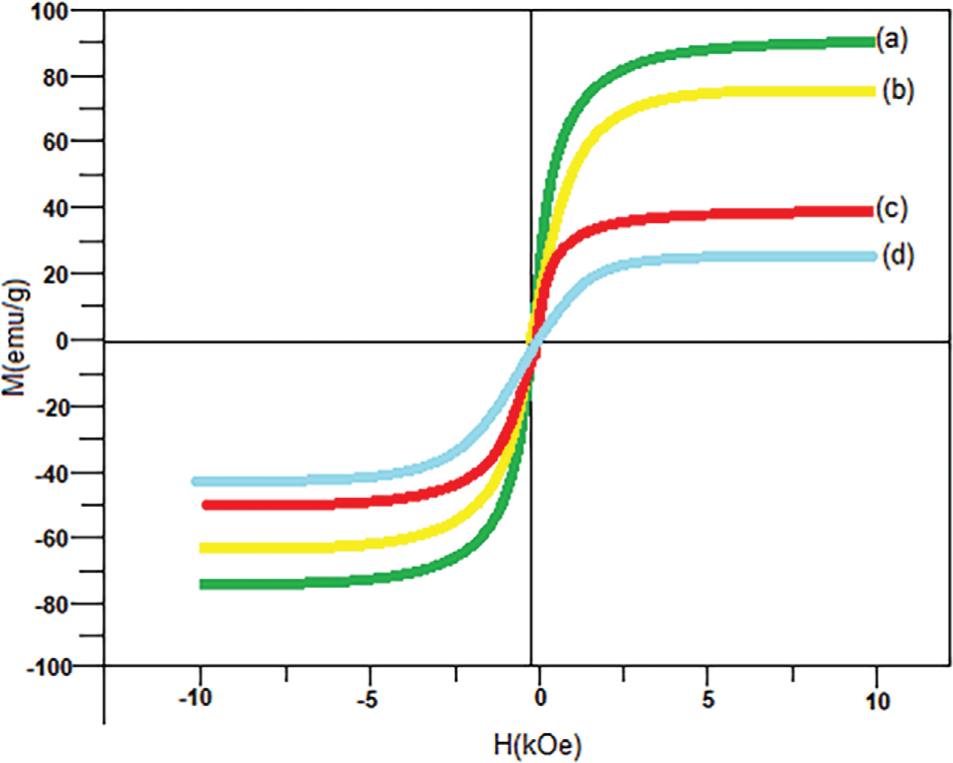
Hysteresis curves of a) Fe_3_O_4_, b) Fe_3_O_4_@SiO_2_, c) Fe_3_O_4_@SiO_2_@ZnO, and d) Fe_3_O_4_@SiO_2_@ZnO–Ag composites.

VSM curves of the synthesized composites are shown in **Figure** **7**. Due to their reuse and magnetic separation, the core–shell structures of Fe_3_O_4_, Fe_3_O_4_@SiO_2_, Fe_3_O_4_@SiO_2_@ZnO, and Fe_3_O_4_@SiO_2_@ZnO–Ag were magnetically investigated. It can be seen from the VSM curves in Figure 7 that all four samples exhibited supermagnetic properties at 300 K with no apparent persistence and no coercivity. First, the magnetization saturation values of Fe_3_O_4_ nanoparticles were measured and determined as 81 emus × g^−1^. Then, the Ms value of Fe_3_O_4_@SiO_2_ spheres covered with SiO_2_ layer was measured and it was seen that the value decreased to 64 emus × g^−1^. Finally, after coating the ZnO and Ag nanoparticles, the Ms values decreased to 41 and 20 emus × g^−1^, respectively. In addition, the Ms value of Fe_3_O_4_@SiO_2_@ZnO–Ag microspheres is high enough to allow effective separation and purification. The decrease in Ms values after coating indicates that the coatings were made successfully.^[^ ^26^, ^29^ ^]^

**Figure 8:**
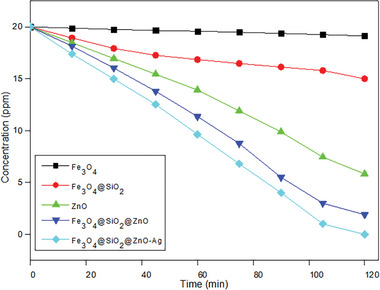
Degradation graph of Fe_3_O_4_, Fe_3_O_4_@SiO_2_, ZnO, Fe_3_O_4_@SiO_2_@ZnO and Fe_3_O_4_@SiO_2_@ZnO–Ag composites on Acid Blue 161 dyestuff.

**Figure** **8** shows the photocatalytic degradation of Fe_3_O_4_, Fe_3_O_4_@SiO_2_, Fe_3_O_4_@SiO_2_@ZnO, and Fe_3_O_4_@SiO_2_@ZnO–Ag composites on Acid Blue 161 dye. Photocatalytic experiments were carried out in a dark environment (0‐15 min) and under UV irradiation (15–120 min). No major change occurred in the photocatalytic degradation of Acid Blue 161, which was carried out in a dark environment. Due to the reactive oxygen species on the surface of Fe_3_O_4_ particles, some dye removal is in question. The dye decomposition rate of Fe_3_O_4_ particles in 120 min is 4.36%. The dye removal of the Fe_3_O_4_@SiO_2_ composite is higher than Fe_3_O_4_ nanoparticles. Within 120 min, the Fe_3_O_4_@SiO_2_ composite removed 11.53% of the dye. Semiconductor ZnO nanoparticles removed 70.8% of the dye, while the ZnO and Ag‐doped Fe_3_O_4_@SiO_2_ composites removed 100% of the dye. The main reason why the Fe_3_O_4_@SiO_2_@ZnO composite shows higher removal than ZnO nanoparticles is the decomposition of the dye as a result of the interaction of the SiO_2_ shell with ZnO. In the synthesis of Fe_3_O_4_@SiO_2_@ZnO by Gohari et al., the effect of calcination temperature on photocatalytic activity was examined at different calcining temperatures of 200, 300, 400, and 500 °C. Accordingly, it was observed that the samples without calcination showed the best photocatalytic activity. Additionally, as the calcination temperature increases, agglomeration and average particle size also increase, resulting in a decrease in the surface area. For these reasons, the calcination of the Fe_3_O_4_@SiO_2_@ZnO composite calcined at 200 °C was carried out at low temperatures to increase the photocatalytic activity efficiency. Photocatalytic degradation begins with the adsorption of Acid Blue 161 dye to the catalyst surface. Moreover, the process of electron photogeneration from the valence band (VB) to the conduction band (CB) is easy when the trigger actively participates in the absorption of radiation, which results in the formation of a positively charged vacancy called vacancies (h+). The photogenerated electron‐hole pairs (e−/h+) are then captured by the surface of the catalyst. Photogenerated electrons react with O2 on the catalyst surface, leading to ROS such as ∙O2− and ∙OH. The interaction of ∙O2 and ∙OH with Acid Blue 161 completely decomposes into non‐toxic inorganic substances and causes complete mineralization of the main compound, CO_2_ and H_2_O.

The degradation mechanism of Acid Blue 161 dye is shown below:

(3) $$\begin{array}{ccc} & & Fe_{3}O_{4}@\mathrm{Si}O_{2}@\mathrm{ZnO}-\mathrm{Ag}+h\nu \to Fe_{3}O_{4}@\mathrm{Si}O_{2}@\mathrm{ZnO}\\ & & \;-\;\mathrm{Ag}\left(ec_{B}-+hv_{B}+\right)\left(2\right)ec_{B}-+O2\to \cdot O2^{-}\left(3\right)hv_{B}++H_{2}O\\ & & \;\to \cdot \mathrm{OH}+H^{+}\left(4\right)\mathrm{Acid}\mathrm{Blue}161\mathrm{dye}+\cdot \mathrm{OH}/\cdot O2^{-}\\ & & \;\to {\mathrm{CO}}_{2}+H_{2}O+\mathrm{other}\;\mathrm{product}\left(5\right) \end{array}$$

**Figure 9:**
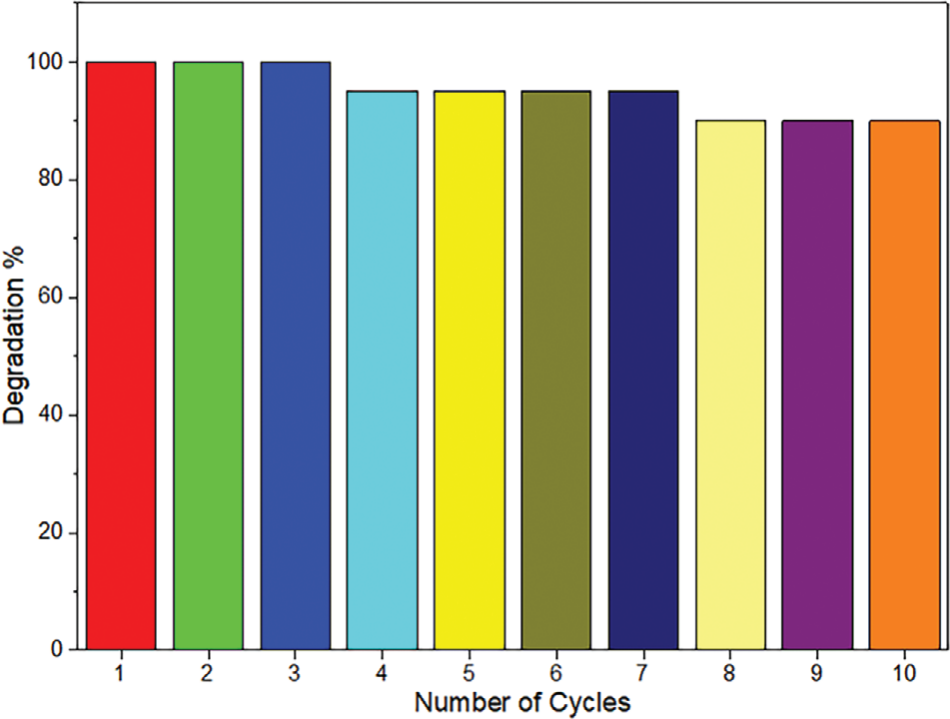
Illustration of the removal percentages of the degradation of Fe_3_O_4_@SiO_2_@ZnO–Ag composite on AB161 after 10 cycles.

After the degradation of Fe_3_O_4_@SiO_2_@ZnO–Ag photocatalyst, Acid Blue 161 dye, the dispersion was filtered, washed with water and ethanol, and dried at room temperature. A new dye solution was then prepared and used again for photochemical degradation. The reusability of the photocatalyst in 10 cycles is shown in **Figure** **9**. It is understood that the efficiency of the photocatalyst is high after 10 cycles for the degradation of AB161 dye under UV light. Experiments determined that the recyclability was around 90% and that the photocatalyst had excellent stability.

The degradation efficiency of Acid Blue 161 dye decreased due to the reuse of the magnetic photocatalyst. As a result of recycling experiments repeated ten times, the degradation rate of Acid Blue 161 decreased to 90%, indicating the reusability of the magnetic photocatalyst. The reusability feature makes the use of magnetic photocatalysts suitable for removing paint wastewater and reducing costs.

**Figure 10:**
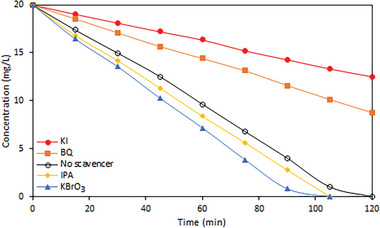
The effect of scavengers on the photocatalytic oxidation process of Acid Blue 161 dyestuff on Fe_3_O_4_@SiO_2_@ZnO–Ag composite.

As shown in **Figure** **10**, to examine the effect of the pollutant on the degradation process, KI, BQ, and KBrO3, inorganic scavengers, and IPA, an organic scavenger, were added to the solution medium. The oxidation reaction of Fe_3_O_4_@SiO_2_@ZnO–Ag composite was carried out using UV light.

According to the test results, the oxidation process remained unchanged with the addition of KBrO3, one of the inorganic scavengers. While a significant change occurred with the addition of BQ, another inorganic scavenger, blockages occurred in the photocatalytic oxidation reaction with the addition of KI inorganic scavenger. With the addition of organic scavenger IPA, no change in the oxidation reaction process was observed. Accordingly, it was determined that voids were used as the primary motivation source in the photocatalytic oxidation process and oxygen peroxide radicals served as the main active species.

Coating with Ag nanoparticles significantly increased the photocatalytic activity of the Fe_3_O_4_@SiO_2_@ZnO composite. This is because Ag nanoparticles accumulate photogenerated electrons and cause the separation of electron‐hole pairs induced by light.^[^ ^1^ ^]^

The photocatalytic efficiency of the prepared composites is as follows:

(4) $$\begin{array}{ccc} & & {\mathrm{Fe}}_{3}O_{4}@\mathrm{Si}O_{2}@\mathrm{ZnO}-\mathrm{Ag}>{\mathrm{Fe}}_{3}O_{4}@\mathrm{Si}O_{2}@\mathrm{ZnO}\\ & & \;>\mathrm{ZnO}\;\mathrm{nanoparticles}>{\mathrm{Fe}}_{3}O_{4}@\mathrm{Si}O_{2}>{\mathrm{Fe}}_{3}O_{4} \end{array}$$

## 3 Conclusion

The core–shell structures of magnetically separable Fe_3_O_4_@SiO_2_@ZnO–Ag composites were characterized using TEM, SEM‐EDS, XRD, FTIR, VSM, XPS, UV–Vis, and their photocatalytic performance on AB161 dye was evaluated. The ZnO shell was coated with Ag nanoparticles to prevent electron‐hole transfer between ZnO and magnetic core particles from reducing photocatalytic activity and to increase photocatalytic efficiency. As a result of TEM and SEM analysis, the diameters of Fe_3_O_4_, Fe_3_O_4_@SiO_2_, Fe_3_O_4_@SiO_2_@ZnO, and Fe_3_O_4_ @SiO_2_@ZnO–Ag composites were determined as 210, 220, 230, and 235 nm. In XRD analysis, the face‐centered cubic structure of Fe_3_O_4_ particles and the hexagonal wurtzite structure of ZnO were determined and it was determined that they were compatible with standard diffraction cards. It was determined that the absorption peaks seen at certain wavelengths were able to synthesize the composites successfully and although there was no specific absorption peak of Ag, Fe_3_O_4_@SiO_2_@ZnO structure affected the shape and density. According to the Debye‐Scherrer formula, the average diameters of Fe_3_O_4_, Fe_3_O_4_@SiO_2_, Fe_3_O_4_@SiO_2_@ZnO, and Fe_3_O_4_@SiO_2_@ZnO–Ag composites were calculated as 207, 212, 238 and 238.8, respectively. According to UV–vis analysis, *E*_g_ values for Fe_3_O_4_, Fe_3_O_4_@SiO_2_, Fe_3_O_4_@SiO_2_@ZnO, and Fe_3_O_4_@SiO_2_@ZnO–Ag composites were found as 1.3, 1.68, 2.21 and 2.15 eV, respectively. As a result of VSM analysis, magnetization values of Fe_3_O_4_, Fe_3_O_4_@SiO_2_, Fe_3_O_4_@SiO_2_@ZnO, and Fe_3_O_4_SiO_2_@ZnO–Ag core–shell microspheres were found as 81, 64, 41 and 20 emu x g^−1^, respectively.

Among the photocatalysts prepared, Fe_3_O_4_@SiO_2_@ZnO–Ag composite Acid Blue 161 shows superior removal and recyclability against photodegradation of the dyestuff. Fe_3_O_4_@SiO_2_@ZnO–Ag composite, which performs better than Fe_3_O_4_@SiO_2_@ZnO and ZnO nanoparticles, is promising in wastewater treatment.

## 4 Experimental Section

### Materials

Iron (III) chloride hexahydrate (FeCl_3_.6H_2_O,97%), sodium acetate (CH3COONa,>99.0%), ethylene glycol (C_2_H_6_O_2_,99.8%), polyethylene glycol (PEG‐H(OCH_2_CH_2_)_n_OH, wt.8000), tetraethyl orthosilicate (TEOS‐Si(OC_2_H_5_)_4_,98%), concentrated ammonia solution (NH_4_OH,25 wt.%), Zinc acetate dihydrate (Zn(Ac)_2_.2H_2_O,>98%), Polyvinylpyrrolidone (PVP‐(C_6_H_9_NO)_n_,wt.40,000), silver nitrate (AgNO_3,_>99.0%), sodium hydroxide (NaOH,97%), ethylalcohol (C_2_H_5_OH,99.8%), and distilled water are used. Acid Blue 161 dye was used for photocatalytic experiments.

### Synthesis of Fe_3_O_4_

By using the solvothermal method, magnetic Fe_3_O_4_ particles were prepared according to our previous research.^[^ ^30^ ^]^ In this synthesis method, FeCl_3_ × 6H_2_0 (5.6 g) was dissolved in ethylene glycol (80 mL) under ultrasonic mixing and mixed until a clear yellow solution was obtained. Sodium acetate (14.4 g) and PEG (4 g) were then added to this solution and further ultrasonic stirred for 15 min. Subsequently, the resulting solution was transferred to a Teflon‐lined stainless steel autoclave reactor and kept in a 200 °C oven for 12 h, and allowed to cool to room temperature. The obtained Fe_3_O_4_ particles were washed several times with ethanol and deionized water and then dried 60 °C for 3 h.

### Synthesis of Fe_3_O_4_@SiO_2_

By using the Stöber method, the Fe_3_O_4_@SiO_2_ composite was prepared according to our previous research.^[^ ^30^ ^]^ Fe_3_O_4_ (0.1 g) particles were added into the solution containing absolute ethanol (80 mL), deionized water (20 mL), and concentrated ammonia (5 mL, 25 wt.%), and mixed under ultrasonication for 15 min. TEOS (1 mL) was then added dropwise to the solution medium under continuous stirring. The obtained Fe_3_O_4_@SiO_2_ composite was washed several times with ethanol and deionized water and then dried 60 °C for 3 h and calcined for 2 h at 200 °C.

### Synthesis of Fe_3_O_4_@SiO_2_@ZnO

The Fe_3_O_4_@SiO_2_ (0.2 g) composite was added to the solution containing ethanol (100 mL), and Zn(Ac)_2_.2H_2_0 (1 g) and mixing ultrasonically until a homogeneous solution obtained at 60 °C for 15 min. Then 0.25 m NaOH (20 mL) solution was added dropwise to the prepared solution and stirred at 60 °C for 4 h at an amplitude of 45%. Then, the solution cooled at room temperature was washed several times with ethanol and deionized water, dried at 60 °C for 3 h, and calcined at 200 °C for 2 h.

### Synthesis of Fe_3_O_4_@SiO_2_@ZnO–Ag

Fe_3_O_4_@SiO_2_@ZnO–Ag composite was synthesized by the simple in situ wet chemistry method. First, Fe_3_O_4_@SiO_2_@ZnO composite (0.4 g) was ultrasonically mixed for 15 min in Tollens' reagent (100 mL, 5×10^−3^ m), to obtain a homogeneous solution.^[^ ^31^ ^]^ After 15 min of mixing, the [Ag(NH_3_)_2_]^+^ ions are adsorbed by the surface of the Fe_3_O_4_@SiO_2_@ZnO composite. Second, PVP (0.6 g) dispersed in ethanol (90 mL) was added into the Fe_3_O_4_@SiO_2_@ZnO solution and refluxed at 70 °C for 4 h. The Fe_3_O_4_@SiO_2_@ZnO–Ag composite was washed several times with ethanol and deionized water and dried at 60 °C for 3 h and calcined at 200 °C for 2 h.

### Characterization

Morphology, size, and elemental analysis of all composites were characterized using scanning electron microscopy (SEM, Zeiss‐Sigma 300), transmission electron microscopy (TEM, Hitachi‐HT7700), and Energy Dispersive X‐ray Spectroscopy (EDS). X‐ray photoelectron spectroscopy analysis was successfully performed using an (XPS, Specs‐Flex). The structure and composition of the composites were examined by X‐ray diffraction (XRD, PANalytical‐Empyrean) using Cu‐Kα radiation with wavelength (λ = 0.15 418 nm). The magnetic behavior of the composites was analyzed using a vibrating sample magnetometer (VSM, Lake Shore, 7407), which is a physical property measurement system applied from −10 to 10 (kOe). The surface functionalization was characterized in Fourier transform infrared (FTIR, Vertex‐80v) spectroscopy using the transmission mode in the 4000–500 cm^−1^ frequency range. UV–vis absorption spectroscopy (UV, Shimadzu‐UV3600 Plus) was used to determine the bandgap energy.

### Photocatalytic Degradation of Acid Blue 161 (AB161)

Photocatalytic performances of catalysts were evaluated by photocatalytic decomposition of Acid Blue 161 dye. Before illumination, the suspension was stirred for 30 min in the dark medium to ensure the adsorption‐desorption balance between the photocatalyst and AB161. Then 100 mg of the photocatalyst was dispersed in 400 mL of dye solution with a dye concentration of 20 ppm. The experiments were carried out in a batch reactor whose outer part was isolated against the light. The temperature of the solution was kept constant at 25 °C with a water circulator. Pen‐Ray UV Lamp (Cole‐Parmer, 257 nm, 44 W.m^−2^) was used as the UV source. After each photocatalytic experiment, the catalyst was separated from the solution with the help of a magnet. Dye concentration was determined using a UV spectrometer (Optizen α spectrophotometer) and concentration measurements were carried out at 602.5 nm.

## Conflict of Interest

The authors declare no conflict of interest.

## Data Availability

The data that support the findings of this study are available from the corresponding author upon reasonable request.
